# Extracting interpretable single-cell metabolic states with graph-guided representation learning

**DOI:** 10.64898/2026.09.17.751504

**Published:** 2026-09-24

**Authors:** Daniel P. Lewinsohn, Nicolas Dias, Adelina Chau, Yuko Koike, Zachary D. Smith, Nilah M. Ioannidis, Allon Wagner

## Abstract

Metabolism shapes cellular function and state, yet measuring single-cell metabolic states at scale remains a challenge. We present Metabolic Representation Net (MeRN), a graph-guided variational autoencoder that leverages prior metabolic knowledge as a topology graph to learn latent representations of metabolic state and reaction activity from single-cell transcriptomes. MeRN’s scalable estimation of reaction activity enables the definition of *data-driven pathways* (DDPs): context-specific metabolic modules supported by transcriptomic evidence and agnostic of standard pathway definitions. Using DDPs, we introduce the *weakest link analysis* to identify metabolic network rewiring. MeRN recovers metabolic zonation in the mouse intestine, links a folate deficiency-induced break in *de novo* purine synthesis to embryonic neural tube defects, shows cytokines with similar non-metabolic effects can elicit divergent T cell metabolism, and identifies metabolic drivers of T cell exhaustion and therapy response in human cancers. Our results establish MeRN as a unified method for metabolic analysis of single-cell transcriptomes.

**Research highlights:**

- MeRN leverages the metabolic topology to comprehensively predict reaction- and pathway-level metabolic activities
- MeRN enables data-driven pathways (DDPs) that capture empirically supported cell-type-specific metabolic modules, agnostic of standard pathway definitions
- MeRN-based DDPs identify metabolic network rewiring of *de novo* purine synthesis due to folate deficiency during embryonic neural tube development
- MeRN identifies human pan-cancer metabolic drivers of T cell exhaustion and Treg-specific metabolic adaptations

## Full Text Availability

The license terms selected by the author(s) for this preprint version do not permit archiving in PMC. The full text is available from the preprint server.

